# Concentrations of IGF-I and IGFBP-3 and pancreatic cancer risk in the European Prospective Investigation into Cancer and Nutrition

**DOI:** 10.1038/bjc.2012.19

**Published:** 2012-02-07

**Authors:** S Rohrmann, V A Grote, S Becker, S Rinaldi, A Tjønneland, N Roswall, H Grønbæk, K Overvad, M C Boutron-Ruault, F Clavel-Chapelon, A Racine, B Teucher, H Boeing, D Drogan, V Dilis, P Lagiou, A Trichopoulou, D Palli, G Tagliabue, R Tumino, P Vineis, A Mattiello, L Rodríguez, E J Duell, E Molina-Montes, M Dorronsoro, J-M Huerta, E Ardanaz, S Jeurnink, P H M Peeters, B Lindkvist, D Johansen, M Sund, W Ye, K-T Khaw, N J Wareham, N E Allen, F L Crowe, V Fedirko, M Jenab, D S Michaud, T Norat, E Riboli, H B Bueno-de-Mesquita, R Kaaks

**Affiliations:** 1 Division of Cancer Epidemiology and Prevention, Institute of Social and Preventive Medicine, University of Zurich, Hirschengraben 84, Zürich 8001, Switzerland; 2 Division of Cancer Epidemiology, German Cancer Research Center (DKFZ), Heidelberg, Germany; 3 Institute of Laboratory Medicine, Clinical Chemistry and Molecular Diagnostics, University Hospital Leipzig, Leipzig, Germany; 4 International Agency for Research on Cancer (IARC-WHO), Lyon, France; 5 Diet, Cancer and Health, Danish Cancer Society, Copenhagen, Denmark; 6 Department of Medicine V, Aarhus University Hospital, Aarhus, Denmark; 7 Department of Epidemiology, School of Public Health, Aarhus University, Aarhus, Denmark; 8 Inserm, Centre for Research in Epidemiology and Population Health, Institut Gustave Roussy, Villejuif, France; 9 Paris South University, Villejuif, France; 10 Department of Epidemiology, German Institute of Human Nutrition, Nuthetal, Germany; 11 Hellenic Health Foundation, Athens, Greece; 12 WHO Collaborating Centre for Food and Nutrition Policies, Department of Hygiene, Epidemiology and Medical Statistics, University of Athens Medical School, Athens, Greece; 13 Bureau of Epidemiologic Research, Academy of Athens, Athens, Greece; 14 Department of Epidemiology, Harvard School of Public Health, Boston, MA, USA; 15 Molecular and Nutritional Epidemiology Unit, Cancer Research and Prevention Institute (ISPO), Florence, Italy; 16 Lombardy Cancer Registry and Environmental Epidemiology Unit, Fondazione IRCCS Istituto Nazionale dei Tumori, Milan, Italy; 17 Cancer Registry and Histopathology Unit, ‘Civile M.P. Arezzo’ Hospital, Ragusa, Italy; 18 School of Public Health, Imperial College London, London, UK; 19 HuGeF Foundation, Torino, Italy; 20 Department of Clinical and Experimental Medicine, Federico II University, Naples, Italy; 21 Public Health and Participation Directorate, Health and Health Care Services Council, Asturias, Spain; 22 Unit of Nutrition, Environment and Cancer, Catalan Institute of Oncology (ICO-IDIBELL), Barcelona, Spain; 23 Andalusian School of Public Health, Granada, Spain; 24 Consortium for Biomedical Research in Epidemiology and Public Health (CIBER) de Epidemiología y Salud Pública (CIBERESP), Spain; 25 Epidemiology and Health Information, Public Health Division of Gipuzkoa, Basque Regional Health Department, San Sebastian, Spain; 26 Department of Epidemiology, Murcia Regional Health Authority, Murcia, Spain; 27 Navarre Public Health Institute, Pamplona, Spain; 28 National Institute for Public Health and Environment (RIVM), Bilthoven, The Netherlands; 29 Department of Gastroenterology and Hepatology, University Medical Centre Utrecht (UMCU), Utrecht, The Netherlands; 30 Julius Center, University Medical Center Utrecht, Utrecht, The Netherlands; 31 Institute of Medicine, Sahlgrenska Academy, University of Gothenburg, Gothenburg, Sweden; 32 Department of Surgery, Skåne University Hospital, SUS, Malmö, Sweden; 33 Departments of Surgical and Perioperative Sciences, Surgery and Public Health and Clinical Medicine, Nutrition Research, Umeå University, Umeå, Sweden; 34 Department of Medical Epidemiology and Biostatistics, Karolinska Institutet, Stockholm, Sweden; 35 The Medical Biobank at Umeå University, Umeå, Sweden; 36 Department of Public Health and Primary Care, University of Cambridge, Cambridge, UK; 37 Medical Research Council (MRC) Epidemiology Unit, Cambridge, UK; 38 Cancer Epidemiology Unit, Nuffield Department of Clinical Medicine, University of Oxford, Oxford, UK; 39 Department of Epidemiology, Division of Biology and Medicine, Brown University, Providence, RI, USA

**Keywords:** IGF-I, IGFBP-3, pancreatic cancer, cohort study

## Abstract

**Background:**

Insulin-like growth factors (IGFs) and their binding proteins (BPs) regulate cell differentiation, proliferation and apoptosis, and may have a role in the aetiology of various cancers. Information on their role in pancreatic cancer is limited and was examined here in a case–control study nested within the European Prospective Investigation into Cancer and Nutrition.

**Methods:**

Serum concentrations of IGF-I and IGFBP-3 were measured using enzyme-linked immunosorbent assays in 422 cases and 422 controls matched on age, sex, study centre, recruitment date, and time since last meal. Conditional logistic regression was used to compute odds ratios (OR) and 95% confidence intervals (CI) adjusted for confounding variables.

**Results:**

Neither circulating levels of IGF-I (OR=1.21, 95% CI 0.75–1.93 for top *vs* bottom quartile, *P*-trend 0.301), IGFBP-3 (OR=1.00, 95% CI 0.66–1.51, *P*-trend 0.79), nor the molar IGF-I/IGFBP-3 ratio, an indicator of free IGF-I level (OR=1.22, 95% CI 0.75–1.97, *P*-trend 0.27), were statistically significantly associated with the risk of pancreatic cancer. In a cross-classification, however, a high concentration of IGF-I with concurrently low levels of IGFBP-3 was related to an increased risk of pancreatic cancer (OR=1.72, 95% CI 1.05–2.83; *P*-interaction=0.154).

**Conclusion:**

On the basis of these results, circulating levels of components of the IGF axis do not appear to be the risk factors for pancreatic cancer. However, on the basis of the results of a subanalysis, it cannot be excluded that a relatively large amount of IGF-1 together with very low levels of IGFBP-3 might still be associated with an increase in pancreatic cancer risk.

Pancreatic cancer is one of the most common causes of cancer deaths in the western world. In Europe, 48 300 deaths in men and 46 900 deaths in women due to pancreas cancer were estimated for 2008 (Ferlay *et al*, 2010). So far, only few risk factors for pancreatic cancer have been clearly identified. Smoking is the major established lifestyle factor known to cause pancreatic cancer, accounting for up to 25–30% of all pancreas cancer cases (Lowenfels and Maisonneuve, 2004). Some nutrition-related factors have also been found to be associated with pancreas cancer risk, including excess body weight (Berrington de Gonzalez *et al*, 2003; Jiao *et al*, 2010), history of type-2 diabetes mellitus (Huxley *et al*, 2005), elevated blood levels of glucose (Gapstur *et al*, 2000; Batty *et al*, 2004; Jee *et al*, 2005; Stolzenberg-Solomon *et al*, 2005; Stattin *et al*, 2007; Grote *et al*, 2011), and possibly, chronic hyperinsulinemia (Stolzenberg-Solomon *et al*, 2005).

Insulin-like growth factors (IGFs) are multifunctional peptides that regulate cell proliferation, differentiation, and apoptosis (Khandwala *et al*, 2000). IGF-I is an important regulator of cell growth in the postnatal period (Khandwala *et al*, 2000). IGF-binding proteins (IGFBP-1 through IGFBP-6) modulate the biological effects of IGF-I, as they determine the concentration of biologically active, unbound IGF (Jones and Clemmons, 1995). More than 90% of circulating IGF-I is bound to IGFBP-3, and less than 1% circulates in free form (Grimberg and Cohen, 2000). Binding of IGF-I to the IGF-I receptor leads to stimulation of mitogenesis in a number of cell types, to cellular protection from apoptosis, and to cellular transformation (Grimberg and Cohen, 2000). IGFBP-3 has growth-inhibiting properties by competitively binding IGF-I, but it also has independent growth inhibiting effects, for example, via induction of apoptosis (Rajah *et al*, 1997, 2002). IGFBP activities are, among others, regulated by IGFBP proteases, which may cleave IGFBPs into fragments with lower affinity to IGFs (Nunn *et al*, 1997).

Ohmura *et al* (1990) have shown that IGF-I can stimulate pancreatic cancer cell growth *in vitro*, and that this effect is mediated by the IGF-I receptor (Bergmann *et al*, 1995). The analysis of pancreatic cancer tissue revealed increased IGF-I mRNA and IGF-I receptor mRNA levels, compared with tissue of healthy individuals (Bergmann *et al*, 1995). Similarly, increased levels of IGF-I and increased IGF-I receptor expression were observed in pancreatic cancer tissue compared with normal pancreas tissue (Karna *et al*, 2002). It appears that IGF-I stimulation and subsequent suppression of tumour suppressor chromosome 10 (PTEN) activity enhance invasiveness and proliferation of the pancreatic cancer cells (Ma *et al*, 2010).

Circulating levels of IGF-1- and IGF-binding proteins have been found to be associated with several types of cancers, including colon (Rinaldi *et al*, 2010), prostate (Roddam *et al*, 2008), and breast cancer (The Endogenous Hormones and Breast Cancer Collaborative Group, 2010). However, the number of studies conducted with respect to pancreatic cancer is limited, as is the number of cases in these studies. The results of the prospective studies are rather inconsistent, however, with most studies showing no association of circulating IGF-I or IGFBP-3 levels with pancreatic cancer risk (Lin *et al*, 2004; Stolzenberg-Solomon *et al*, 2004; Wolpin *et al*, 2007; Douglas *et al*, 2010). Because of the inconsistencies of previous studies, we examined the association between IGF-I, IGFBP-3, and pancreatic cancer in the prospective European Prospective Investigation into Cancer and Nutrition (EPIC) cohort, including more than 400 incident cases of pancreatic cancer.

## Material and methods

### Study description

European Prospective Investigation into Cancer and Nutrition is a prospective cohort study that includes more than 500 000 male and female participants recruited in 23 centres in 10 European countries between 1992 and 2000. Most centres recruited subjects from the general population, but in Utrecht and Florence, only women from breast cancer screening programs were recruited; the Spanish and Italian centres include blood donors, and the French cohort consists of members of a health insurance for state school employees. A high proportion of participants of the Oxford cohort are vegetarians or health-conscious volunteers. The cohorts of France, Utrecht, Florence, and Norway include women only.

Information on lifestyle and diet was collected during baseline examination. Diet was assessed using country-specific, validated dietary assessment instruments (Kaaks *et al*, 1997; Riboli and Kaaks, 1997). Information on smoking, alcohol consumption, physical activity, education, occupation, and medical and reproductive history was collected using questionnaires and personal interviews. Anthropometric measurements was conducted during the baseline examination (Haftenberger *et al*, 2002).

Following a standardized protocol, a blood sample of 30 ml was collected in all participating EPIC countries. In all centres except Oxford, blood samples were stored protected from light at 5–10°C until further processing and aliquoting. In the Oxford centre, blood samples were collected throughout the United Kingdom and transported to the laboratory in Norfolk by mail at an ambient temperature. In all centres except Denmark and Sweden, 0.5–1.5 ml aliquots of serum, plasma, red blood cells, and buffy coat were filled into plastic straws and stored in liquid nitrogen at −196°C. In the Danish centres, 1 ml aliquots were filled into tubes and stored in the vapour phase of liquid nitrogen containers (−150°C). In Sweden, the samples were stored at −80°C.

### Selection of case and control subjects

Pancreatic cancer incidence data were coded according to ICD-10, and included all invasive exocrine pancreatic cancers that were coded as C25 (25.0–25.3, 25.7–25.9). Cases were those EPIC participants who developed pancreatic cancer after their recruitment into the cohort and before the end of the study period. Individuals were excluded when diagnosed with another malignant tumour before the diagnosis of pancreatic cancer, except for non-melanoma skin cancer, and when no blood specimens were available for analysis. A total of 638 incident cases of pancreatic cancer occurred until December 2006; 578 of them were primary exocrine pancreatic tumours. Blood specimens were available for 422 of these cases. The included 422 pancreatic cancer cases were similar in their characteristics to the overall 578 cases with pancreatic adenocarcinoma (data not shown). Of the 422 cases, 307 (76%) were microscopically confirmed. The remaining 24% were diagnosed by clinical symptoms, imaging results, or physical examination. Forty-one percent of the tumours occurred in the head of the pancreas, followed by body (7%) and tail (5%); the rest of the tumours were of unknown localization. One control, alive and free of cancer at time of diagnosis of the index case, was selected for each case using incidence density sampling, that is, controls may include subjects who became a case later in time, and each control may be sampled more than once. Cases and controls were matched by study centre, sex, age at enrolment (±6 months), date of entry in the cohort, time between blood sampling and time of last consumption of foods and drinks (<3 h, 3–6 h, >6 h).

### Laboratory assays

Serum IGF-I and IGFBP-3 concentrations were measured in the immunoassay laboratory at the German Cancer Research Center (DKFZ), Heidelberg, Germany. Both peptides were analysed by enzyme-linked immunosorbent assays purchased from Beckman Coulter (Webster, TX, USA). Before the total IGF-I analysis, IGF-I was separated from IGF-I-binding proteins by an acid–ethanol extraction step. Cases and matched controls were measured in singleton within the same batch. Each analytical batch further included three different serum quality control samples. Laboratory staff were blinded to the case/control status of the study samples. Intra-batch and inter-batch coefficients of variation for IGF-I and IGFBP-3 were 12.8 and 12.9%, and 6.5 and 7.2%, respectively.

### Statistical analysis

Conditional logistic regression was used to examine the associations of IGF-I and IGFBP-3 concentrations with pancreatic cancer risk. We also computed the molar ratio of IGF-I to IGFBP-3 (IGF-I/IGFBP-3 ratio) as a marker of the estimated level of IGF-I biologically available to bind to its receptor. Concentrations of IGF-I and IGFBP-3, as well as IGF-I/IGFBP-3 ratio were categorized into sex-specific quartiles, based on the distribution among all controls. Crude models took into account matching criteria; multivariate models were additionally adjusted for body mass index (continuous), smoking history (never, former, quitting smoking less than 10 years ago, more than 10 years ago, current, with 1–9, 10–19, or ⩾20 cigarettes per day, missing), and history of diabetes (self-reported or high glycated haemoglobin (HbA1c) concentration (⩾6.5%)). These covariates were added in the multivariable adjusted models, because they were associated with pancreatic cancer, correlated with IGF-1 or IGFBP-3, or changed the logistic *β*-estimate by more than 10%. Further adjustment was made for circulating C-peptide concentration, which has been measured previously on the same subjects (Grote *et al*, 2011). Further analyses were conducted with mutual adjustments between IGF-I and IGFBP-3 concentrations.

Sub-analyses were performed, stratified by sex, smoking status at baseline (smoker/non-smoker), diabetes (defined by self-report or HbA1c concentrations (⩾6.5%), waist circumference (</⩾median; 96 cm for men and 80 cm for women), length of follow-up (⩽/>2 year of follow-up time in cases), concentration of C-peptide (</⩾ median, 5.57 ng ml^−1^), and microscopical verification of cases. Odds ratios (OR) were estimated for quartiles of IGF-1 and IGFBP-3 concentrations, as well as IGF-I/IGFBP-3 ratio. Additionally, we examined the interaction between IGF-I and IGFBP-3 levels (both variables were dichotomized by median concentration) in a cross-classification. Statistical tests for heterogeneity were based on *χ*^2^-statistics, calculated as the deviations of logistic beta-coefficients observed in each of the subgroups, relative to the overall beta-coefficient. All analyses were conducted with SAS version 9.2 (Cary, NC, USA).

## Results

Of the 422 cases in this analysis, 46% were men (Table 1). Mean age at baseline was 58 years; mean age at diagnosis was 63 years. Female cases had a higher body mass index and waist circumference than female controls, but no difference was observed among men. Cases were more often smokers at baseline than controls, and they more often reported a diagnosis of diabetes at baseline or had elevated baseline blood levels of HbA1c. IGF-1 was not statistically significantly correlated with body mass index (Pearson's partial correlation coefficient; adjusted for age, sex, and study centre; *r*=−0.07 (95% confidence intervals (CI) −0.17 to 0.03)), waist circumference (*r*=−0.03 (95% CI −0.13 to 0.08)), or circulating C-peptide level (*r*=0.10 (95% CI −0.005 to 0.20)), and rather weakly with height (*r*=0.11 (95% CI 0.01 0.21)), whereas IGFBP-3 showed correlations with body mass index (*r*=0.12 (95% CI 0.01 to 0.22)), waist circumference (*r*=0.13 (95% CI 0.03 0.23)), and C-peptide levels (*r*=0.20 (95% CI 0.10 0.30)), but not with height (*r*=0.01 (95% CI −0.09 to 0.12)). IGF-1 correlated highly and significantly with IGFBP-3 (*r*=0.52 (95% CI 0.44 0.59)) and the molar IGF-1/IGFBP-3 ratio (*r*=0.78 (95% CI 0.74 0.82)), whereas the ratio showed no correlation with IGFBP-3 (*r*=−0.09 (95% CI −0.19 to 0.01)).

Circulating levels of IGF-I or IGFBP-3 were not related to the risk of pancreatic cancer (Table 2). Using molar IGF-I/IGFBP-3 ratio as an indicator of free IGF-I concentration, we also observed no association with pancreatic cancer risk. The results were only slightly affected by different types of adjustment. Additional mutual adjustment of IGF-I and IGFBP-3 also did not strongly change the observed associations with pancreatic cancer. There were also no associations of IGF-I, IGFBP-3, or the ratio of these two with pancreatic cancer, when using only microscopically confirmed cases (Table 3).

In sub-analyses, we examined whether the association of IGF-I, IGFBP-3, or IGF-I/IGFBP-3 with pancreatic cancer was modified by sex, smoking status, length of follow-up, waist circumference, diabetes status, or circulating C-peptide concentration (Table 3). With few exceptions, we did not observe statistically significant heterogeneity between subgroups. Waist circumference modified the association of IGF-1 and IGF-1/IGFBP-3 ratio with pancreatic cancer risk, such that IGF-1 concentration were positively associated with pancreatic cancer in subjects with low waist circumference when comparing quartile 3 with quartile 1; no association was observed when comparing the top *vs* the bottom quartile. There were no statistically significant associations among individuals with waist circumference above the median.

We cross-classified the IGF-I and IGFBP-3 concentration and observed an increased risk of pancreatic cancer among those with IGF-I concentration above the median and IGFBP-3 concentration below the median (OR=1.72, 95% CI 1.05–2.83); however, the test for interaction was not statistically significant (*P*=0.154; Table 4).

## Discussion

We examined the association of components of the IGF axis in association with the risk of pancreatic cancer in the largest prospective study, so far without finding any indication for an association with the circulating levels of IGF-I and IGFBP-3. There was, however, an increased risk among those with high IGF-I and concurrently low IGFBP-3 concentrations, although the interaction was not statistically significant. Evans *et al* (1997) found no elevated serum levels of IGF-I and IGFBP-3 in pancreatic cancer compared with controls. In contrast, Karna *et al* (2002) showed significant increases in serum IGF-I and IGFBP-3 levels in patients with pancreatic cancer compared with control subjects. Among prospective studies, a case–control study nested within the ATBC trial did not observe associations of serum concentrations of IGF-1, IGFBP-3, or IGF-1/IGFBP-3 ratio with the risk of pancreatic cancer (Stolzenberg-Solomon *et al*, 2004); however, this result is based on a cohort of male smokers only. This null association, though, is similar to the results seen in the four US cohorts that were analysed together (Wolpin *et al*, 2007). Only a nested case–control study conducted in Japan reported that individuals in the highest quartile of IGF-I concentration had an OR of 2.31 (95% CI 0.70–2.64) compared with participants in the lowest quartile (Lin *et al*, 2004). A recently published study nested in the PLCO cohort observed an increased risk of pancreatic cancer with increasing IGF-I/IGFBP-3 molar ratio, which was interpreted as an indicator of the concentration of free IGF-I (Douglas *et al*, 2010). We did not observe an increase in risk with increasing IGF-I/IGFBP-3 molar ratio, but did observe that those participants with high IGF-I levels above the median and low IGFBP-3 concentrations had an increased risk of pancreatic cancer.

IGFBP-3 is supposed to have growth-inhibiting properties and one would, thus, expect that high IGFBP-3 concentrations are inversely associated with cancer risk. On the other hand, IGFBP-3 and IGF-I are highly correlated in our cohort. In the Japanese nested case–control study, IGFBP-3 concentration was positively associated with pancreatic cancer risk; the risk of death from pancreatic cancer was increased with increasing levels of serum IGFBP-3, with the OR for the highest quartile being 2.53 (95% CI=0.93–6.85; Lin *et al*, 2004). However, the results of previous studies on different types of cancer have been inconsistent with some studies indeed showing inverse associations, but some also showing no or even a positive association (Renehan *et al*, 2004). Cleavage of IGFBPs by proteases results in IGFBP fragments with lower affinity to IGFs and additionally influences IGF-I bioavailability by reducing the amount of functional IGFBPs. It has been suggested that the maintenance of normal IGFBP levels is critical to normal rates of cell growth and cell death (Nunn *et al*, 1997; Firth and Baxter, 2002). It has also been discussed that different assays measuring concentrations of total or intact IGFBP-3 could cause differences between studies (Kaaks *et al*, 2001; Renehan *et al*, 2004; Rinaldi *et al*, 2005). We measured intact IGFBP-3, not total IGFBP-3, which also includes IGFBP-3 fragments that are less biologically active.

Most IGF-I in the circulation is produced by the liver (Pollak *et al*, 2004). A major factor stimulating the hepatic production of IGF-I and IGFBP-3 is growth hormone (Jones and Clemmons, 1995), but insulin also has a central role in regulating levels of IGF-I and IGFBPs. For example, insulin increases the hepatic levels of growth hormone receptors, thereby enhancing liver synthesis of IGF-I, and in addition, insulin increases bioactivity of IGF-I by inhibiting the synthesis of IGFBP-1 (Lee *et al*, 1997; Kaaks and Lukanova, 2001). Therefore, we stratified our analysis by circulating C-peptide concentration to evaluate whether the association between IGF-1 and IGFBP-3, and pancreatic cancer was different in individuals with high or low C-peptide levels. However, we observed no statistically significant interaction, which is similar to what Wolpin *et al* (2007) had observed in their analysis. We also examined whether other factors that are either well-known risk factors for pancreatic cancer or are associated with IGF-I concentration modified the effect of IGF-I or IGFBP-3 on pancreatic cancer risk. However, none of the factors examined modified the observed association. We only observed statistically significant interactions of waist circumference with levels of IGF-I or IGF-I/IGFBP-3 ratio, but the associations in the respective subgroups were not consistently statistically significant.

In the EPIC cohort, only one blood sample has been collected at baseline. It might be that repeated measurements of IGF-I and IGFBP-3 more accurately reflect circulating levels at different points in time. However, single serum measurements of IGF-I and IGFBP-3 generally have been found to be quite representative of serum concentrations over longer time periods. In a study within the New York University Women's Health Study cohort, Spearman's rank correlations between repeat measurements in serum samples collected over time periods of more than 1 year were 0.87 and 0.73, respectively, for IGF-I and IGFBP-3 (Lukanova *et al*, 2004). Other research groups have reported similar levels of reproducibility for circulating IGF-I and IGFBP-3 (Goodman-Gruen and Barrett-Connor, 1997; Chan *et al*, 1998).

In conclusion, our results generally do not support the hypothesis that circulating levels of IGF-1 and IGFBP-3, or the molar IGF-I/IGFBP-3 ratio are associated with the risk of pancreatic cancer, which confirms the results of most previous prospective studies. However, it is noteworthy that individuals with high circulating IGF-I and low IGFBP-3 levels have an increased risk of pancreatic cancer, compared with those with low IGF-I and high IGFBP-3 concentrations.

## Figures and Tables

**Table 1:** Baseline characteristics of pancreatic cancer cases and matched controls

**Variable**	**Cases (*n*=422)**	**Controls (*n*=422)**
Male subjects, n (%)	195 (46)	195 (46)
Age at recruitment (y), mean (range)	58 (30–76)	58 (30–76)
Age at diagnosis (y), mean (range)	63 (37–82)	—
Follow-up (y), mean (range)	5.4 (0–13)	
		
*BMI (kg m* ^*−2*^ *), mean±s.d.*		
Male	26.88±3.6	26.7±3.7
Female	26.5±4.9	25.2±4.2
		
*Height (cm), mean±s.d.*		
Male	174.6±7.4	175.1±7.7
Female	162.3±6.6	161.5±7.2
		
*Waist circumference (cm), mean±s.d.*		
Male	96.2±10.1	96.6±10.3
Female	84.3±12.3	81.1±10.6
		
*Smoking status*, n *(%)*^a^		
Never	155 (37)	189 (45)
Former	130 (31)	137 (32)
Current	132 (31)	91 (22)
		
*Education*, n *(%)*^a^		
Primary school or less	165 (40)	158 (39)
University	82 (20)	86 (21)
		
*Physical activity, n* *(%)*^a^		
Active	62 (16)	60 (16)
Inactive	103 (27)	119 (31)
		
*Alcohol intake at recruitment (g per day), mean±s.d.*		
Male	19.7±24.4	20.4±26.2
Female	9.1±13.1	7.4±10.6
		
*Fasting status*, n *(%)*		
Fasting (⩾6 h)	117 (28)	113 (27)
In between (3–6 h)	158 (37)	163 (39)
Non-fasting (<3 h)	66 (16)	66 (15)
Unknown	81 (19)	80 (19)
Self-reported diabetes at recruitment, n *(%)*	30 (7)	17 (4)
Subjects HbA1c ⩾6.5%, n (%)	50 (12)	28 (7)
C-peptide (ng ml^−1^), mean±s.d.	6.98±4.6	6.66±4.5
IGF-1 (ng ml^−1^), mean±s.d.	184.8±71.3	182.5±68.5
Male	187.1±74.1	185.7±68.3
Female	182.9±68.9	179.7±68.7
		
IGFBP-3 (ng ml^−1^), mean±s.d.	4668±1209	4665±1085
Male	4411±1267	4484±1042
Female	4890±1114	4821±1100
		
IGF-1/IGFBP-3 ratio	0.19±0.06	0.18±0.06

Abbreviations: BMI=body mass index; IGF=insulin-like growth factor; IGFBP=IGF-binding protein; IQR=interquartile range; y=years.

a Percentages do not add up to 100%, because not all subgroups are shown.

**Table 2:** Relative risk (OR (95% CI)) of pancreatic cancer by quartiles of IGF-1, IGFBP-3, and its ratio in EPIC

	**Quartiles^a^**				**OR**		
	**1**	**2**	**3**	**4**	***P*-trend^b^**	**Continuous**	***P*-trend^c^**
*IGF-1 men (ng ml^−1^)*	33–138	139–176	177–226	227–437	—	Per 10 ng ml^−1^	—
*IGF-1 women (ng ml^−1^)*	40–128	129–168	169–220	221–433	—	—	—
Number ofcases/controls	103/104	88/105	115/106	114/105	—	—	—
Model 1^d^	1.0	0.88 (0.58–1.31)	1.17 (0.76–1.81)	1.21 (0.75–1.93)	0.301	1.01 (0.98–1.04)	0.499
Model 2^e^	1.0	0.88 (0.58–1.34)	1.21 (0.77–1.90)	1.14 (0.70–1.85)	0.475	1.01 (0.98–1.04)	0.526
Model 3^f^	1.0	0.88 (0.58–1.35)	1.23 (0.78–1.94)	1.15 (0.70–1.88)	0.469	1.01 (0.98–1.04)	0.460
Model 4^g^	1.0	0.86 (0.55–1.35)	1.21 (0.74–1.98)	1.13 (0.67–1.92)	0.542	1.01 (0.98–1.04)	0.721
Model 5^h^	1.0	0.89 (0.56–1.42)	1.27 (0.75–2.14)	1.21 (0.66–2.25)	0.439	1.01 (0.97–1.05)	0.597
							
*IGFBP-3 men (ng ml^−1^)*	1625–3800	3825–4548	4550–5057	5092–9367	—	Per 100 ng ml^−1^	—
*IGFBP-3 women (ng ml^−1^)*	1698–4085	4087–4740	4745–5321	5342–11 128	—	—	—
Number of cases/controls	114/104	107/106	84/106	116/105	—	—	—
Model 1^d^	1.0	0.90 (0.60–1.36)	0.72 (0.48–1.08)	1.00 (0.66–1.51)	0.789	1.00 (0.99–1.01)	0.954
Model 2^e^	1.0	1.00 (0.65–1.52)	0.75 (0.49–1.14)	1.13 (0.73–1.74)	0.812	1.00 (0.99–1.02)	0.593
Model 3^f^	1.0	1.04 (0.67–1.59)	0.76 (0.49–1.17)	1.06 (0.68–1.65)	0.941	1.00 (0.99–1.01)	0.970
Model 4^g^	1.0	1.05 (0.67–1.65)	0.81 (0.52–1.27)	1.07 (0.67–1.71)	0.969	1.00 (0.99–1.02)	0.696
Model 5^h^	1.0	1.00 (0.63–1.59)	0.76 (0.47–1.23)	0.94 (0.55–1.61)	0.673	1.00 (0.98–1.02)	0.875
							
*IGF-I/IGFBP-3 men*	0.05–0.15	0.16–0.18	0.19–0.22	0.22-0.43	—	Per 0.01	—
*IGF-I/IGFBP-3 women*	0.05–0.12	0.13–0.16	0.17–0.21	0.22–0.44	—	—	—
Number of cases/controls	103/104	88/105	112/105	116/105	—	—	—
Model 1^d^	1.0	0.87 (0.57–1.33)	1.12 (0.73–1.72)	1.22 (0.75–1.97)	0.273	1.02 (0.99–1.05)	0.190
Model 2^e^	1.0	0.86 (0.55–1.33)	1.10 (0.70–1.70)	1.12 (0.68–1.85)	0.480	1.01 (0.98–1.04)	0.425
Model 3^f^	1.0	0.96 (0.61–1.52)	1.21 (0.77–1.90)	1.29 (0.77–2.16)	0.245	1.02 (0.99–1.05)	0.207
Model 4^g^	1.0	0.94 (0.59–1.51)	1.14 (0.71–1.84)	1.17 (0.69–2.00)	0.467	1.01 (0.98–1.04)	0.594

Abbreviations: BMI=body mass index; CI=confidence interval; EPIC=European Prospective Investigation into Cancer and Nutrition; IGF=insulin-like growth factor; IGFBP=IGF-binding protein; OR=odds ratio.

a Quartile cut points were based on the distribution of controls.

b *P*-trend test was based on median values of each quartile.

c *P*-trend test was based on continuous values.

d Model 1: logistic regression conditioned on matching factors (EPIC recruitment centre, sex, age at recruitment, date at entry in the cohort, time between blood sampling and last consumption of foods and drinks).

e Model 2: as model 1 with further adjustment for smoking (never, former, quitting smoking less than 10 years ago, more than 10 years ago, current, with 1–9, 10–19, or ⩾20 cigarettes per day, missing).

f Model 3: as above with further adjustment for BMI (continuous) and diabetes (defined by self-report or HbA1c concentration ⩾6.5%).

g Model 4: as above with further adjustment for C-peptide concentration (continuous).

h Model 5: as above with further adjustment for IGFBP-3 concentration (continuous).

i Model 5: as model 3 with further adjustment for IGF-I concentration (continuous).

**Table 3:** Relative risk (OR (95% CI)) of pancreatic cancer by quartiles of IGF-1, IGFBP-3, and its ratio for subgroups in EPIC

	**No**	**Quartiles^a^**					
	**Ca/Co**	**1**	**2**	**3**	**4**	***P*-trend^b^**	***P*-interaction**
*IGF-1 men (ng ml^−1^)*	195/195	33–138	139–176	177–226	227–437	—	—
*IGF-1 women (ng ml^−1^)*	225/227	40–128	129–168	169–220	221–433	—	—
							
Non-smoker	154/189	1.0	1.09 (0.57–2.07)	1.63 (0.85–3.13)	1.72 (0.91–3.28)	0.068	0.445
Smokers	261/228	1.0	0.73 (0.42–1.26)	0.98 (0.57–1.68)	0.94 (0.54–1.64)	0.878	—
							
Non-diabetics	349/374	1.0	0.88 (0.56–1.37)	1.23 (0.80–1.91)	1.20 (0.77–1.88)	0.244	0.545
Diabetics	54/32	1.0	1.54 (0.40–5.99)	0.69 (0.14–3.32)	0.81 (0.18–3.75)	0.629	—
							
Years 1 and 2 of follow-up	71/71	1.0	0.59 (0.17–2.04)	1.09 (0.29–4.09)	1.43 (0.36–5.69)	0.417	0.946
3+ years of follow-up	349/351	1.0	0.93 (0.58–1.48)	1.26 (0.77–2.08)	1.22 (0.71–2.11)	0.391	—
							
Male	195/195	1.0	0.71 (0.37–1.35)	1.18 (0.59–2.37)	1.09 (0.53–2.23)	0.553	0.746
Female	225/227	1.0	1.09 (0.60–1.98)	1.28 (0.68–2.42)	1.08 (0.52–2.25)	0.827	—
							
Low C-peptide	214/221	1.0	0.64 (0.37–1.12)	0.85 (0.48–1.51)	1.08 (0.59–1.95)	0.513	0.398
High C-peptide	206/201	1.0	1.15 (0.61–2.14)	1.57 (0.87–2.84)	1.27 (0.71–2.29)	0.396	—
							
Low waist circumference	205/221	1.0	0.83 (0.46–1.52)	1.80 (1.01–3.23)	1.23 (0.68–2.23)	0.246	0.040
High waist circumference	215/201	1.0	0.79 (0.44–1.41)	0.70 (0.38–1.27)	1.03 (0.56–1.86)	0.812	—
							
Microscopically confirmed	305/307	1.0	0.95 (0.57–1.58)	1.45 (0.83–2.52)	1.10 (0.61–2.00)	0.632	—
							
*IGFBP-3 men (ng ml^−1^)*	195/195	1625–3800	3825–4548	4550–5057	5092–9367	—	—
*IGFBP-3 women (ng ml^−1^)*	227/226	1698–4085	4087–4740	4745–5321	5342–11 128	—	—
							
Non-smoker	155/189	1.0	1.00 (0.53–1.89)	0.77 (0.40–1.47)	0.94 (0.50–1.79)	0.701	0.974
Smokers	262/227	1.0	0.94 (0.55–1.59)	0.73 (0.42–1.26)	1.05 (0.62–1.77)	0.987	—
Non-diabetics	351/373	1.0	1.02 (0.67–1.55)	0.69 (0.44–1.08)	0.99 (0.64–1.52)	0.622	0.694
Diabetics	54/32	1.0	0.61 (0.13–2.82)	1.13 (0.25–5.16)	1.12 (0.27–4.61)	0.797	—
							
Years 1 and 2 of follow-up	71/70	1.0	1.68 (0.59–4.78)	1.36 (0.44–4.23)	1.44 (0.46–4.47)	0.612	0.724
3+ years of follow-up	351/351	1.0	0.94 (0.58–1.52)	0.69 (0.43–1.13)	1.12 (0.68–1.85)	0.855	—
							
Male	195/195	1.0	0.78 (0.41–1.46)	0.54 (0.28–1.04)	0.96 (0.50–1.84)	0.774	0.362
Female	227/226	1.0	1.37 (0.74–2.56)	1.03 (0.56–1.90)	1.17 (0.61–2.25)	0.915	—
							
Low C-peptide	215/221	1.0	0.78 (0.45–1.35)	0.66 (0.37–1.16)	0.64 (0.36–1.14)	0.088	0.198
High C-peptide	207/200	1.0	1.21 (0.66–2.23)	0.78 (0.42–1.45)	1.47 (0.82–2.65)	0.277	—
							
Low waist circumference	206/221	1.0	0.74 (0.43–1.30)	0.57 (0.32–1.02)	0.92 (0.51–1.63)	0.632	0.654
High waist circumference	216/200	1.0	1.26 (0.69–2.27)	0.91 (0.49–1.68)	1.05 (0.59–1.87)	0.825	—
							
Microscopically confirmed	307/306	1.0	0.98 (0.57–1.66)	0.65 (0.38–1.12)	0.94 (0.55–1.62)	0.624	—
							
*IGF-I/IGFBP-3 men*	195/195	0.05–0.15	0.16–0.18	0.19–0.22	0.22–0.43	—	—
*IGF-I/IGFBP-3 women*	225/226	0.05–0.12	0.13–0.16	0.17–0.21	0.22–0.44		
							
Non-smoker	154/189	1.0	1.08 (0.56–2.11)	1.54 (0.81–2.92)	1.83 (0.94–3.57)	0.052	0.683
Smokers	261/227	1.0	0.81 (0.47–1.39)	1.03 (0.60–1.75)	1.16 (0.67–1.99)	0.442	—
							
Non-diabetics	349/373	1.0	1.02 (0.65–1.59)	1.27 (0.82–1.97)	1.39 (0.89–2.17)	0.097	0.543
Diabetics	54/32	1.0	0.28 (0.06–1.29)	1.00 (0.25–4.08)	0.91 (0.18–4.59)	0.923	—
							
Years 1 and 2 of follow-up	71/70	1.0	1.23 (0.26–5.75)	2.05 (0.38–11.08)	0.85 (0.18–4.03)	0.761	0.513
3+ years of follow-up	349/351	1.0	1.01 (0.62–1.63)	1.20 (0.75–1.94)	1.45 (0.83–2.54)	0.163	—
							
Male	195/195	1.0	0.49 (0.24–0.98)	1.07 (0.55–2.08)	1.28 (0.62–2.64)	0.161	0.050
Female	225/226	1.0	1.45 (0.76–2.77)	1.31 (0.67–2.55)	1.18 (0.52–2.65)	0.856	—
							
Low C-peptide	214/221	1.0	0.64 (0.36–1.16)	0.91 (0.50–1.64)	1.20 (0.67–2.17)	0.254	0.423
High C-peptide	206/200	1.0	1.42 (0.78–2.60)	1.33 (0.77–2.32)	1.35 (0.75–2.45)	0.370	—
							
Low waist circumference	205/221	1.0	0.85 (0.46–1.56)	1.73 (0.94–3.17)	1.33 (0.73–2.39)	0.162	0.028
High waist circumference	215/200	1.0	1.01 (0.56–1.81)	0.75 (0.43–1.30)	1.35 (0.74–2.48)	0.491	—
							
Microscopically confirmed	305/306	1.0	1.25 (0.72–2.18)	1.52 (0.88–2.61)	1.49 (0.81–2.72)	0.195	—

Abbreviations: BMI=body mass index; CI=confidence interval; EPIC=European Prospective Investigation into Cancer and Nutrition; IGF=insulin-like growth factor; IGFBP=IGF-binding protein; OR=odds ratio.

a Logistic regression conditioned on matching factors (EPIC recruitment centre, sex, age at recruitment, date at entry in the cohort, time between blood sampling and last consumption of foods and drinks) and adjusted for smoking (never, former, quitting smoking less than 10 years ago, more than 10 years ago, current, with 1–9, 10–19, or ⩾20 cigarettes per day, missing), BMI (continuous) and diabetes (defined by self-report or HbA1c concentrations ⩾6.5%).

b *P*-trend test was based on median values of each quartile.

**Table 4:** Joint effect of IGF-1 and IGFBP-3 concentrations on risk of pancreatic cancer (OR (95% CI))a,^b^

	**<Median IGF-1**	**⩾Median IGF-1**
⩾Median IGFBP-3	1.0	1.48 (0.97–2.22)
<Median IGFBP-3	1.47 (0.97–2.22)	1.72 (1.05–2.83)

Abbreviations: BMI=body mass index; CI=confidence interval; EPIC=European Prospective Investigation into Cancer and Nutrition; IGF=insulin-like growth factor; IGFBP=IGF-binding protein; OR=odds ratio.

a Logistic regression conditioned on matching factors (EPIC recruitment centre, sex, age at recruitment, date at entry in the cohort, time between blood sampling and last consumption of foods and drinks) and adjusted for smoking (never, former, quitting smoking less than 10 years ago, more than 10 years ago, current, with 1–9, 10–19, or ⩾20 cigarettes per day, missing), BMI (continuous) and diabetes (defined by self-report or HbA1c concentrations ⩾6.5%).

b *P*-interaction=0.154.
